# Advanced Electrochemical Sensors for Rapid and Sensitive Monitoring of Tryptophan and Tryptamine in Clinical Diagnostics

**DOI:** 10.3390/bios15090626

**Published:** 2025-09-19

**Authors:** Janani Sridev, Arif R. Deen, Md Younus Ali, Wei-Ting Ting, M. Jamal Deen, Matiar M. R. Howlader

**Affiliations:** 1 Department of Electrical and Computer Engineering, McMaster University, 1280 Main Street West, Hamilton, ON L8S 4K1, Canadaalim129@mcmaster.ca (M.Y.A.);; 2 Department of Integrated Biomedical Engineering and Health Sciences, McMaster University, 1280 Main Street West, Hamilton, ON L8S 4K1, Canada; 3 AI Atlas Inc., Dundas, ON L9H 6J5, Canada; arif.r.deen@outlook.com; 4 Electronics and Communication Engineering Discipline, Khulna University, Khulna 9208, Bangladesh; 5 Department of Chemical Engineering, National Taiwan University of Science and Technology, No. 43 Keelung Road Section 4, Taipei 106, Taiwan; 6 Taiwan Building Technology Center, National Taiwan University of Science and Technology, No. 43 Keelung Road Section 4, Taipei 106, Taiwan; 7 Département BEL, Centre CMP, Mines Saint-Etienne, 880 Route de Mimet, F-13541 Gardanne, France

**Keywords:** electrochemical sensors, tryptophan, tryptamine, nanomaterials, point-of-care diagnostics, artificial intelligence

## Abstract

Tryptophan (Trp) and tryptamine (Tryp), critical biomarkers in mood regulation, immune function, and metabolic homeostasis, are increasingly recognized for their roles in both oral and systemic pathologies, including neurodegenerative disorders, cancers, and inflammatory conditions. Their rapid, sensitive detection in biofluids such as saliva—a non-invasive, real-time diagnostic medium—offers transformative potential for early disease identification and personalized health monitoring. This review synthesizes advancements in electrochemical sensor technologies tailored for Trp and Tryp quantification, emphasizing their clinical relevance in diagnosing conditions like oral squamous cell carcinoma (OSCC), Alzheimer’s disease (AD), and breast cancer, where dysregulated Trp metabolism reflects immune dysfunction or tumor progression. Electrochemical platforms have overcome the limitations of conventional techniques (e.g., enzyme-linked immunosorbent assays (ELISA) and mass spectrometry) by integrating innovative nanomaterials and smart engineering strategies. Carbon-based architectures, such as graphene (Gr) and carbon nanotubes (CNTs) functionalized with metal nanoparticles (Ni and Co) or nitrogen dopants, amplify electron transfer kinetics and catalytic activity, achieving sub-nanomolar detection limits. Synergies between doping and advanced functionalization—via aptamers (Apt), molecularly imprinted polymers (MIPs), or metal-oxide hybrids—impart exceptional selectivity, enabling the precise discrimination of Trp and Tryp in complex matrices like saliva. Mechanistically, redox reactions at the indole ring are optimized through tailored electrode interfaces, which enhance reaction kinetics and stability over repeated cycles. Translational strides include 3D-printed microfluidics and wearable sensors for continuous intraoral health surveillance, demonstrating clinical utility in detecting elevated Trp levels in OSCC and breast cancer. These platforms align with point-of-care (POC) needs through rapid response times, minimal fouling, and compatibility with scalable fabrication. However, challenges persist in standardizing saliva collection, mitigating matrix interference, and validating biomarkers across diverse populations. Emerging solutions, such as AI-driven analytics and antifouling coatings, coupled with interdisciplinary efforts to refine device integration and manufacturing, are critical to bridging these gaps. By harmonizing material innovation with clinical insights, electrochemical sensors promise to revolutionize precision medicine, offering cost-effective, real-time diagnostics for both localized oral pathologies and systemic diseases. As the field advances, addressing stability and scalability barriers will unlock the full potential of these technologies, transforming them into indispensable tools for early intervention and tailored therapeutic monitoring in global healthcare.

## 1. Introduction

Tryptophan (Trp) is an essential aromatic amino acid that serves as the biochemical precursor for a variety of critical metabolites, including serotonin (SR), melatonin, kynurenine (Kyn), and tryptamine (Tryp). These metabolites regulate a wide spectrum of physiological functions ranging from mood regulation and circadian rhythm to immune modulation and metabolic balance [1,2,3]. Dysregulation of Trp metabolism has been implicated in major neuropsychiatric disorders, cancer, metabolic syndrome, and neurodegenerative diseases, underlining its central role as a diagnostic and therapeutic biomarker [1,4]. Among its downstream products, Tryp functions as a neuromodulator and has been associated with mood disorders and schizophrenia, further highlighting its clinical relevance [5,6]. The global burden of mental illness, estimated at 418 million disability-adjusted life years (DALYs) and costing approximately USD 5 trillion annually, emphasizes the urgent need for the sensitive and real-time monitoring of biomarkers such as Trp and Tryp to support early diagnosis, prognosis, and treatment development [7].

Traditional laboratory-based analytical platforms, including high-performance liquid chromatography (HPLC), fluorescence spectroscopy, and enzyme-linked immunosorbent assays (ELISA), remain the gold standards for the accurate quantification of Trp and its metabolites [8,9,10,11]. However, these techniques are constrained by high costs, labor-intensive workflows, extended processing times, and the requirement for specialized personnel and infrastructure. These limitations restrict their use in real-time or decentralized diagnostic settings. In contrast, electrochemical sensors have emerged as a practical and transformative alternative. Offering rapid response, high sensitivity, portability, and cost-effectiveness, they are well suited for direct analysis in complex biological matrices such as serum, saliva, and urine [12,13]. Their compatibility with miniaturization and integration into portable platforms further strengthens their potential for point-of-care (POC) applications [14,15,16,17].

The electrochemical detection of Trp and Tryp, however, is fundamentally constrained by the intrinsic characteristics of their redox chemistry. Oxidation of the indole moiety involves an irreversible two-electron and two-proton transfer, leading to the formation of reactive indolequinone intermediates [11,12]. This process is kinetically sluggish and requires a high overpotential when performed on unmodified electrodes, thereby compromising sensitivity and introducing overlapping signals with structurally related interferents. To address these bottlenecks, recent research has focused on rational electrode engineering, particularly through material innovations and surface modifications. Carbon-based nanomaterials such as graphene (Gr) derivatives and carbon nanotubes (CNTs), as well as hybrid composites incorporating metal nanoparticles, conducting polymers, and metal-organic frameworks (MOFs), have shown remarkable promise. These materials improve electron transfer kinetics, lower overpotentials, and enhance both selectivity and operational stability [14,15,16,17].

In addition to material innovation, advances in device architecture have further improved sensor performance. Flexible substrates and microfluidic integration allow for adaptable designs that enable continuous monitoring in complex biological environments. Moreover, the incorporation of machine learning algorithms for signal deconvolution and data analysis has enhanced the ability of sensors to operate in real time while minimizing the impact of matrix interference [18,19]. Together, these innovations represent a shift from proof-of-concept studies toward practical applications of electrochemical sensors in clinical and industrial contexts.

Previous reviews in this field have generally been limited in scope, often classifying sensors by electroanalytical technique, focusing on specific aspects such as enantiomeric discrimination, or using polymer-based designs [20,21,22,23,24]. While valuable, these narrower perspectives do not fully capture the breadth of recent advances in sensor materials, architectures, and translational applicability. In contrast, the present review provides a comprehensive synthesis of progress achieved over the past decade, highlighting the synergistic role of materials innovation, surface functionalization, and device integration in advancing the electrochemical detection of Trp and Tryp. Particular emphasis is placed on how these innovations improve sensitivity, selectivity, and reproducibility in real biological matrices. Furthermore, this work identifies remaining challenges, including interference from structurally similar compounds, sensor fouling, and the translation of laboratory prototypes to clinically validated devices.

The structure of this review is as follows: Section 2 details the biochemical structures and physiological importance of Trp and Tryp, including their biosynthetic pathways and disease associations. Section 3 surveys traditional laboratory methods for their detection, underscoring their strengths and inherent limitations. Section 4 provides an in-depth analysis of recent electrochemical sensor innovations, organized by material class and architectural strategy, and evaluates their performance in terms of sensitivity, selectivity, and matrix compatibility. Section 5 discusses the role of Trp metabolites in systemic diseases, with particular emphasis on their links to periodontal disease. Finally, Section 6 outlines ongoing research challenges and proposes future directions, including the integration of artificial intelligence (AI) for advanced data analysis, the development of multi-omics sensor platforms, and strategies to bridge the translational gap from laboratory prototypes to clinically validated devices.

By synthesizing interdisciplinary advances in material science, electrochemistry, biomedical engineering, and data analytics, this review provides a roadmap for accelerating the development of robust, accessible, and clinically relevant biosensors for Trp and Tryp. In doing so, it seeks to underscore the unique novelty of these sensors, not only as analytical tools, but also as transformative platforms for precision medicine.

## 2. Trp and Tryp Biomarkers

**Figure 1:**
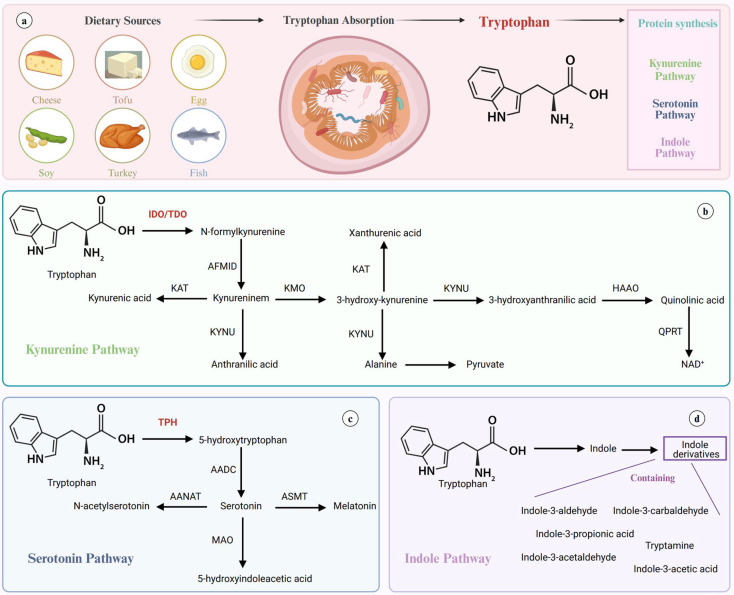
(**a**) Tryp, an essential amino acid from foods such as turkey, eggs, cheese, tofu, seeds, and fish, enters the bloodstream for protein synthesis and metabolism. It is catabolized via three main pathways: (**b**) the Kyn pathway, producing metabolites including Kyn and NAD^+^; (**c**) the SR pathway, yielding SR and melatonin; and (**d**) the indole pathway, where gut microbiota generate indole derivatives. Reprinted with the permission from Ref. [27].

Trp is an amino acid which cannot be synthesized in the body and is supplemented in diet through foods such as turkey, milk and tuna. It has two enantiomers, L- and D-Trp, of which only L-Trp can cross the blood-brain barrier and is utilized in protein synthesis [25]. L-Trp has a significant role in biological processes, whereas D-Trp is less impactful in the body [26]. It is the least abundant amino acid and often acts as a rate-limiting step in its primary role of protein synthesis [25]. The recommended intake of Trp is about 3.5–6.0 mg/kg of body weight per day [25]. Trp has many important metabolites, such as Kyn SR and Trp which are produced from three primary pathways—the Kyn, the SR, and the indole pathways—as shown in Figure 1 [27].

Trp consists of an a-amino group, an a-carboxylic acid group, and a side-chain indole, which is a bicyclic ring formed by benzene and a pyrrole group attached to the a-carbon by a -CH_2_-group [28]. The indole endows Trp with hydrophobic properties and can provide stability through Van der Waals forces. Trp is found in many biofluids and its expression in these biofluids has many implications in the progression of many diseases, such as mood disorders [29]. Trp levels in biofluids and in various conditions are shown in Table 1.

The breakdown of the indole ring produces Kyn, which accounts for 90% of Trp catabolism and is a precursor to kynurenic and quinolinic acid (QA), which are important in the regulation of certain neurotransmitters [34]. The accumulation of Kyn can result in oxidative cell damage and thus inflammation. The other 10% of Trp derivatives maintain the indole ring in the structure and act in chemical messaging pathways, including SR and Tryp. While only 1% of dietary Trp is utilized in brain SR, it is still significant to many important psychological processes. It is heavily implicated in mood disorders such as depression due to its role in adaptive reaction and response to changes in environment [28]. A low Trp diet was used to study the impact of SR on mood and found [25]. SR also is significant in gut function, immune responses, and hemodynamics [28]. This pathway produces melatonin, which is critical for sleep regulation. Tryp is an important neuromodulator of SR and has intrinsic properties independent of SR function.

Tryp or indole-3-ethylamine is an indole compound with an electrophilic substituent in the pyrrole ring [5]. It is formed by the decarboxylation of Trp and is found in trace amounts in mammalian brain tissue [5,35]. It is a neurotransmitter and a neuromodulator responsible for controlling the release of SR and dopamine (DA) [2]. The excessive accumulation of Tryp can affect blood pressure, nausea, and other such complications [17,35]. There is also evidence of Trp implications in disorders such as schizophrenia, bipolar disorder, and obesity [17].

Trp has many implications in processes related to aging. The most significant example is found in the Kyn pathway, specifically in the biological aging biomarker potential of the Kyn/Trp ratio [36]. Age-related chronic inflammation seems to cause an increase in the production of Kyn, leading to an increased ratio. This has been associated with decreased cognitive performance and increased risk of cardiovascular disease. Evidence suggested that the increased ratio is not a by-product of onset of disease, but is intrinsic to the aging process. High levels of Trp also prevent the decline in SR production that is often associated with aging. The decline in SR has been shown to have an impact on cognitive processes and is especially crucial for long-term memory formation [36]. Detection of Trp and its metabolites is crucial for understanding its role as a biological marker for aging and its involvement in cognitive and mood-related disorders in the aging population

## 3. Current Methods of Trp and Tryp Detection

Trp detection is challenging due to its unique properties that separate it from other amino acids. A big challenge is that the standard acidic condition used for the characterization of amino acids triggers the oxidative degeneration of Trp [37]. A solution to this problem is to use alkaline hydrolysis, using sodium hydroxide or other similar alkaline compounds [37]. This method is time-consuming and the largest drawback to this method is the formation of molecular oxygen that interferes with sensing performance. The use of antioxidants, such as ascorbic acid, prior to protein analysis can prevent the degradation of Trp during alkaline hydrolysis [37]. Tryp has been detected with many of the same methods as Trp [38]. They have been characterized through high-performance liquid chromatography (HPLC), liquid chromatography-tandem mass spectrometry, capillary electrophoresis, and optical detection methods [39].

Chromatography separates compounds through studying the interactions between the analyte and a particular solvent and identifying the separate components through these unique interactions [40]. It is often used in conjunction with UV, fluorescence, or mass spectrometry detection. Liquid chromatography has many shortcomings as it is quite slow and has many limiting factors related to the size of the device [40]. HPLC utilizes high pressure to drive the separation and is sensitive and easy to use; however, it can be tedious and have low stability. There are also issues with selectivity in hydrophobic compounds, which is of significance as Trp has hydrophobic properties [40].

Mass spectrometry is typically used to separate electrically charged species in their gas phases [41]. A mass analyzer is used to sort the ions by mass-charge ratios, which are then detected by an ion detector. The results are shown as histograms which present the number of ions at different ratios. This is often used in conjunction with liquid chromatography [38,41]. The combined method allows for high sensitivity and accuracy, while being a fast method of detection. Capillary electrophoresis uses electrophoretic mobility, which is based on charge, viscosity, and the atom’s radius, to separate ions [42]. The applied voltage only triggers the movement of ions and since it is based on both size and charge, it is easy to distinguish between different species. It is a fast, clear means of detection; however, it is not accessible for use in point-of-care settings.

Optical detection, such as the colorimetric method, has also been used to detect Trp. In this method, Trp was converted to indole, which was able to react to form a pink-colored product [20]. While this method was simple and specific, it had poor performance in terms of LOD and linear range (LR). The largest drawback to this method is that it requires spectrophotometric measurement, which is expensive. However, a low-cost alternative, scanometry, has been proposed, which also has the added advantage of improving the clarity of the results [20]. Another colorimetric method based on surface-enhanced Raman scattering (SERS) and the diazo coupling reaction was used, which was able to create a selective sensor. However, highly toxic chemicals were used in this process, which means the sensor cannot be used with patients.

Electrochemical sensors use electrical energy to detect the interaction between an analyte and the sensor surface [13]. They are simple, rapid, low-cost, sensitive, and compatible with complementary metal-oxide semiconductor (CMOS) technology, making them ideal for compact POC devices. Electrochemical sensing techniques can be categorized by their specific techniques, experimental conditions, and output signals. Some of these techniques include cyclic voltammetry (CV), linear sweep voltammetry (LSV), differential pulse voltammetry (DPV), square wave voltammetry (SWV), amperometry, potentiometry, and electrochemical impedance spectroscopy (EIS). Cyclic voltammetry (CV) sweeps the potential of an electrode linearly with time, first in one direction and then in the reverse direction [43]. This technique is useful for studying the redox behavior of molecules; however, its sensitivity is limited by the nonfaradaic current produced by the electric double-layer due to the continuous sweep. In contrast, LSV is a straightforward technique that sweeps the potential only in the forward direction, from a starting potential to a second potential. However, it is less sensitive and not ideal for analyzing redox behaviors. Second derivative linear sweep voltammetry (SDLSV), a subcategory of LSV, plots the second derivative of the current with respect to the potential, which helps to reduce background noise and distinguish closely spaced redox peaks [44]. DPV and SWV are highly sensitive techniques due to their ability to cancel out nonfaradaic currents. They are widely used in the sensing of Trp and Tryp [17,45,46]. In DPV, a staircase waveform is applied, and the difference in current at the beginning and end of each pulse is plotted against voltage. The difference in current between these two points helps to improve sensitivity and reduce background noise. In SWV, a square wave potential is added to a staircase waveform. The current is measured at the end of both the forward and reverse pulses, and the difference is plotted against voltage, enhancing resolution and sensitivity. Amperometry applies a constant potential to an analyte and plots the current over time [47]. It is highly sensitive and easy to implement due to the use of single potential, but lacks selectivity as it combines the current from all electrochemical reactions. Electrochemical sensors have a large scope for sensor material modifications to allow for the enhancement of sensing performances, such as the trace-level detection of Trp, which make them the ideal candidate for the development of an optimal Trp/Tryp sensor [17,48]. They are easily modifiable through surface functionalization, which can improve sensing performance, and are mainly characterized by an LOD, wide LR, and good selectivity and sensitivity.

## 4. Electrochemical Sensors for Trp and Tryp

The electrochemical sensing of Trp showed an irreversible oxidation reaction that conforms to a two-electron/two-proton process [14]. Trp oxidation on an unmodified electrode is often impractical due to the slow electron transfer rate and high overpotential. More importantly, common interferents such as tyrosine (Tyr) have a similar oxidation peak potential to Trp, which causes poor sensor selectivity [48]. To achieve better sensing performances, a variety of surface modifications were performed on bare electrodes and tested in a variety of real samples. The most common substrates used were carbon-based, such as the glassy carbon electrode (GCE), screen-printed carbon electrode (SPCE), and carbon paste electrode (CPE). Common modification materials included carbon derivatives such as multi-walled carbon nanotubes (MWCNTs), reduced graphene oxides (rGOs), and metallic nanoparticles such as gold nanoparticles (AuNPs).

In addition to electrode modification strategies, considerable attention has been given to the standardization of biological samples such as serum, saliva, and milk, due to their inherent enzymatic and biomolecular complexity. Across the reviewed studies, several common pretreatment protocols were employed to minimize variability. pH levels were typically stabilized using phosphate or acetate buffers to preserve the electrochemical activity of Trp and Tryp. Proteins were frequently precipitated using organic solvents such as acetonitrile or methanol, followed by centrifugation to remove residues that might otherwise adsorb irreversibly to electrode surfaces [49]. Dilution with buffers was also widely applied to reduce ionic strength and suppress nonspecific adsorption [50,51]. To control enzymatic activity, rapid cooling, chemical denaturation, or measurement within short storage windows was implemented. Collectively, these pretreatment methods improved both reproducibility and selectivity in Trp and Tryp detection from complex biofluids.

### 4.1. Trp-Sensing Electrodes Modified with Metal Nanoparticles

AuNPs remain among the most effective modifiers for Trp-sensing electrodes due to their high surface-to-volume ratio, electrical conductivity, and chemical stability [52,53,54]. Critically, their function extends beyond reducing detection limits: AuNPs enhance electron transfer kinetics, amplify redox signals, and stabilize immobilized recognition elements in aptamer-based designs. In aptamer (Apt)-functionalized systems, AuNPs increase the stability of redox-active centers, achieving a far superior sensitivity to conventional nanoparticle-modified electrodes [52,53].

**Figure 2:**
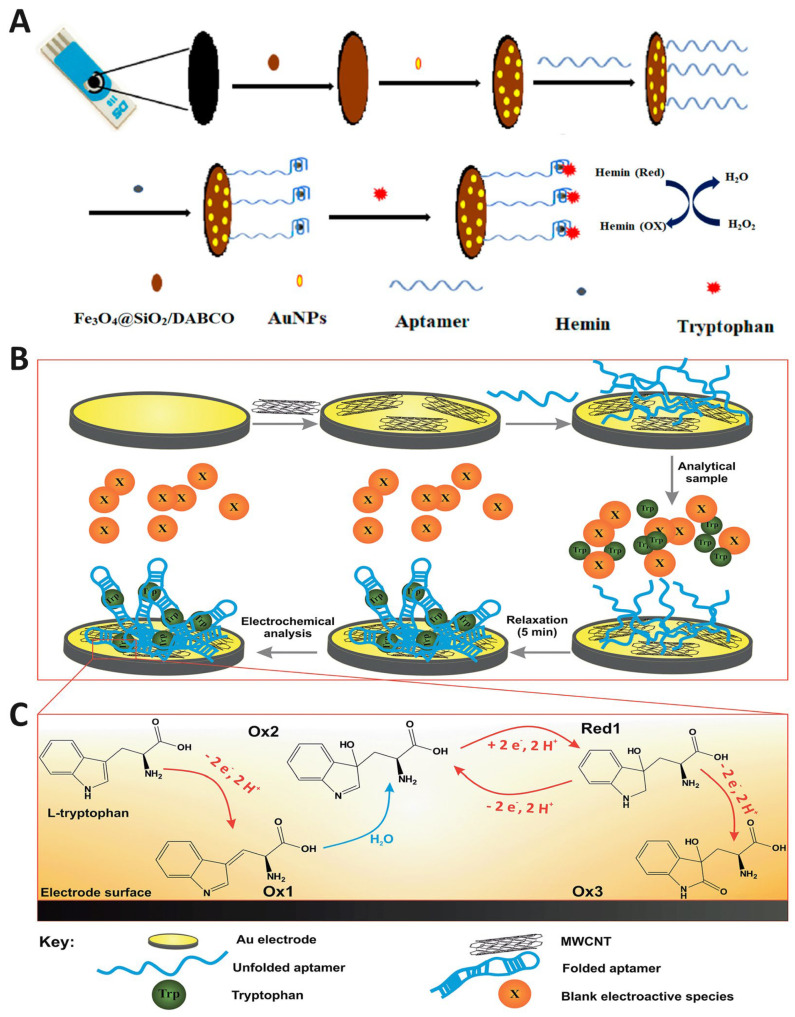
(**A**) Scheme for the fabrication of a gold−based magnetic electrochemical Trp aptasensor. Adopted with permission from Ref. [53]. Copyright MDPI, 2020. (**B**) Fabrication process of the Apt-functionalized multi-walled carbon nanotube gold electrode (Apt−MWCNTs/AuE) for Trp detection. (**C**) Proposed electrochemical oxidation pathway of Trp at the aptasensor surface, showing the sequential transformation from Trp to intermediate oxidation products (Ox_1_−Ox_3_) and corresponding reduced forms (red_1_). The scheme highlights the role of Apt-mediated molecular recognition in facilitating selective preconcentration and signal amplification. Reproduced with permission from Ref. [49]. Copyright Royal Chemicals Society, 2016.

Performance is further enhanced by hybridization strategies. For instance, combining AuNPs with dendritic gold nanostructures (DGNs) or Fe_3_O_4_@SiO_2_/DABCO hybrids (Figure 2A) expands electroactive surface area and lowers charge transfer resistance (*R_ct_*), while mitigating nanoparticle aggregation [53]. Similarly, integration with polydopamine nanospheres (PDNs) improves electrode biocompatibility and stability, while enabling the multiplexed detection of dopamine (DA), uric acid (UA), ascorbic acid (AA), and Trp [54].

Majidi et al. directly compared AuNP-based sensors by developing two configurations: (i) a gold screen-printed electrode (AuSPE) modified with multi-walled carbon nanotubes (MWCNTs) and (ii) an Apt-functionalized electrode integrating MWCNTs and AuNPs (AuE/MWCNTs/Apt) (Figure 2B,C) [49,55]. Using constant-current potentiometric stripping analysis (CC-PSA), the Apt platform achieved a detection limit of 4.9 pM, significantly surpassing the non-Apt electrode (LOD = 360 pM). However, in complex matrices such as serum, saliva, urine, and milk, the conventional AuSPE/MWCNTs sensor performed comparably to, or even slightly better than, the Apt variant. This suggests that while Apt functionalization improves selectivity in controlled conditions, non-specific binding and fouling in biological samples may offset those gains [49].

These studies collectively demonstrate that AuNPs offer unique versatility: they serve as platforms for functionalization rather than as passive modifiers. Hybrid AuNP-based electrodes balance sensitivity, selectivity, and stability, but their performance is ultimately determined by rational design choices. The critical lesson is that translational viability depends less on achieving ultralow LODs in the buffer, and more on reproducibility, stability, and antifouling in complex biofluids.

The summarized comparisons in Table 2 reinforce this conclusion: AuNP-based sensors excel not only because of their intrinsic properties, but because they provide a flexible scaffold for molecular recognition, hybrid material integration, and signal amplification.

Recent studies have demonstrated that metal and metal-oxide nanoparticles, beyond gold, play a pivotal role in enhancing the electrochemical sensing of Trp [59,60,61]. Table 3 summarizes the metal and/or metal-oxide-modified electrodes developed for Trp sensing. For instance, silver-doped titanium dioxide nanoparticles (Ag-TiO_2_ NPs) electrodeposited on GCE exhibited improved electrocatalytic performance due to their sponge-like porous structure and spherical morphology, which significantly increased the effective surface area and facilitated electron shuttling between the electrolyte and electrode interface [59]. This modification not only reduced the kinetic barrier for electron transfer, but also enhanced sensitivity (0.9150 µA/µM) and achieved an ultralow detection limit of 0.003 µM, with successful applicability in food samples such as egg whites. Mechanistically, the presence of Ag dopants induced higher crystallite aggregation and surface roughness, both of which synergistically promoted electron mobility and catalytic activity.

Similarly, UV-irradiated WO_3_ nanoparticles were reported to undergo a reduction in crystallite size and generate oxygen vacancies, features that directly contribute to enhanced catalytic ability by increasing active sites for electron transfer. The resulting WO_3_/GCE sensor enabled the simultaneous determination of Trp with ultralow detection limits and distinct oxidation potentials, highlighting its potential for real-world applications in food matrices such as milk and egg [60]. The mechanistic implication here is that oxygen vacancies serve as electron traps, lowering the overpotential required for amino acid oxidation and thereby facilitating sensitive detection. In another approach, ZnO nanorods electrostatically fused with polythiophene (PT) formed a nanocomposite (PT@ZnO) with high porosity and effective π-π and hydrogen-bonding interactions between the indole moiety of Trp and the sulfur/amine groups in PT. This interaction accelerated electron transfer kinetics and enabled a detection limit of 8.5 nM across a broad concentration range (100 nM–1 mM) [61]. The superior selectivity observed suggests a strong molecular recognition mechanism beyond simple surface adsorption, further supported by its reproducibility in detecting Trp in peanut extract.

### 4.2. Trp-Sensing Electrodes Modified with Carbon Derivatives

Gr and CNTs are among the most widely used electrodes in this class owing to their exceptional electrical conductivity, large surface area, and tunable chemistry [63]. Functionalization and heteroatom doping (N, S, B) change the local electronic structure and can lower overpotentials for particular reactions. Also, many carbon materials can be engineered to be more resistant to fouling than some bare metals, but fouling is highly case-dependent (material form, coatings, and biofluid). Improved resistance to biofouling is highly advantageous for testing in real biofluids.

However, pristine Gr is limited in electrochemical sensing due to its high production costs and strong aggregation via π–π stacking and van der Waals interactions [64]. rGO, derived from graphene oxide (GO), partially restores conductivity and provides electroactive sites, making it widely applicable in sensing platforms [65,66]. Combining rGO with metal oxides or nanoparticles enhances catalytic activity, surface area, and selectivity for Trp detection (Table 4).

TiO_2_/rGO nanocomposites have been applied to Trp sensing, with sodium borohydride modification improving the electrochemically active surface area and electron transfer, resulting in increased anodic peak currents [70]. MnCo_2_O_4_-rGO/GCE sensors exhibit strong electrocatalytic activity due to their multiple oxidation states in MnCo_2_O_4_ and abundant active sites in rGO, providing low detection limits and wide LRs [71]. Metal composites such as Pd–Cu@Cu_2_O/N-rGO further leverage nitrogen-doping to enhance conductivity, increase binding sites, and prevent nanoparticle aggregation, enabling sensitive Trp detection in biological fluids [46].

**Figure 3:**
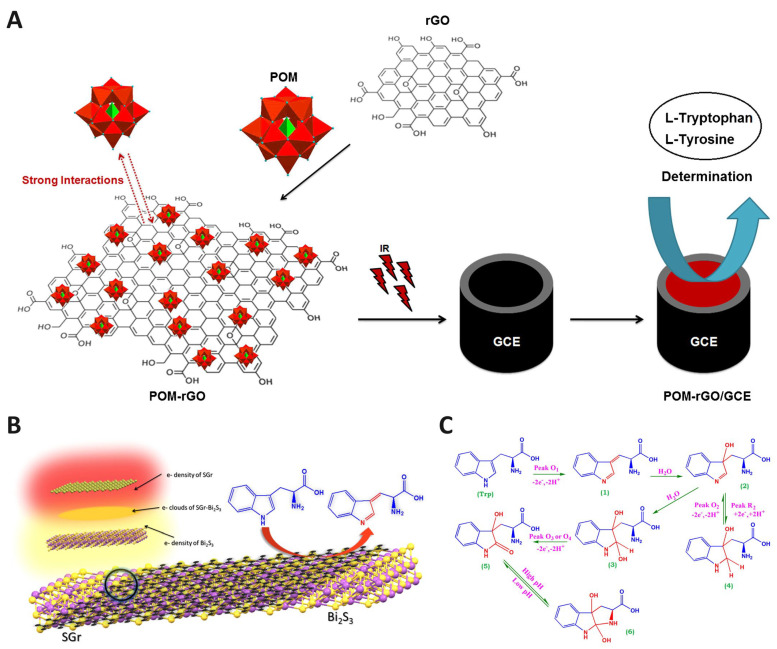
(**A**) Schematic of the fabrication process of POM-rGO/GCE. Adopted with the permission from Ref. [16]. Copyright Elsevier B.V., 2016. (**B**) The heterostructured single crystalline rod of SGr-Bi_2_S_3_, and (**C**) the mechanism of Trp electro-oxidation. Reproduced with permission from Ref. [68]. Copyright Elsevier B.V., 2018.

Spinel-type composites, including N-rGO/CuCo_2_O_4_, offer simultaneous detection of neurotransmitters and Trp, demonstrating high selectivity and stability [72]. Functionalization with graphene quantum dots and bimetallic nanoparticles (rAu–PtNPs/GQDs) or polyoxometalates (POM-rGO) enhances electron transfer and sensitivity, reaching sub-nanomolar limits of detection (Figure 3A) [16,69]. Similarly, silver nanoparticle-decorated GO (AgNPs/GO) and sulfide-doped graphene/Bi_2_S_3_ (SGr-Bi_2_S_3_) highlight the synergistic interplay between conductive carbon matrices and catalytically active nanomaterials, where edge-plane exposure and optimized surface orientation play critical roles in enhancing sensitivity (Figure 3B) [67,68]. These improvements are consistent with the plausible mechanism of Trp electro-oxidation, which involves proton-coupled electron transfer pathways facilitated by the modified electrode surface, as shown in Figure 3C.

Overall, rGO-based composite electrodes significantly improve Trp sensing through enhanced electron transfer, increased surface area, and selective molecular interactions. A critical examination of the literature suggests that the most effective strategies involve combining rGO with catalytically active metals or doped nanostructures while carefully tuning surface chemistry and orientation for real-sample applicability.

### 4.3. Trp-Sensing Electrodes Modified with Carbon-Metal Hybrids

Hybrids that combine carbon nanostructures (e.g., carbon nanofibers (CNFs), MWCNTs, and carbon quantum dots (CQDs)) with transition-metal nanoparticles (Fe, Co, Mn, etc.) are often used in electrocatalysis and sensing because they unite the high surface area, excellent electronic conductivity, and chemical robustness of the carbon scaffold with the redox/catalytic activity of the metal sites [77]. However, their performance under repeated electrochemical cycles can be limited by metal nanoparticle aggregation and metal leaching, since these degradation pathways degrade activity and selectivity over time.

**Figure 4:**
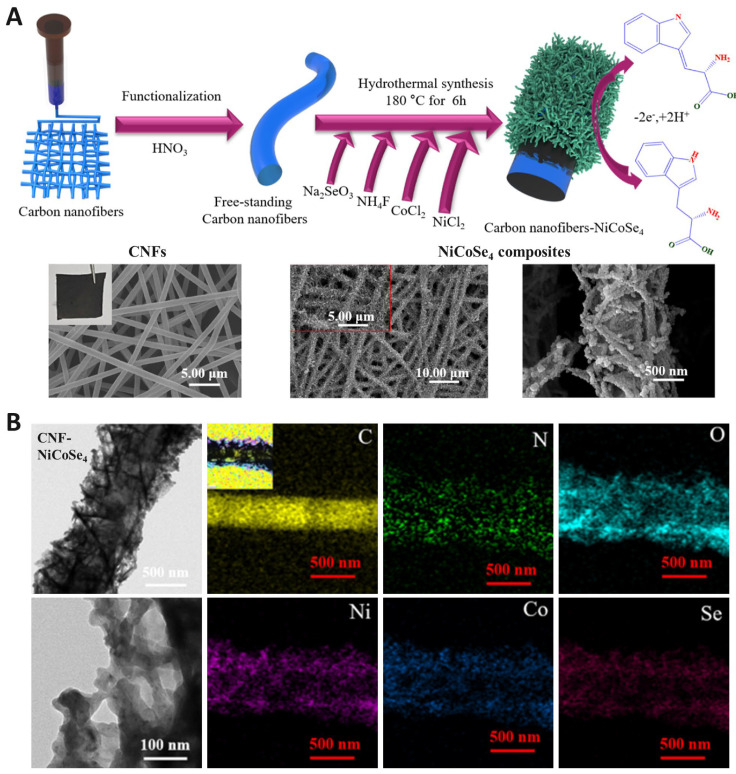
(**A**) Scheme of the fabrication of the CNF-NiCoSe_4_ composites via hydrothermal method. (**B**) Transmission electron microscopy images of the CNF-NiCoSe_4_ composites and the corresponding cross-bonded elemental mapping. Adopted with permission from Ref. [47]. Copyright Elsevier B.V., 2021.

The integration of these two classes of materials often yields hybrid electrodes with enhanced sensitivity and selectivity for Trp detection (Table 5). A consistent feature across multiple studies is that the carbon scaffold supplies defect-rich surfaces and efficient π-π interactions with the indole moiety of Trp, whereas the metallic component facilitates electron-proton transfer and minimizes surface fouling. For example, NiCoSe_4_ nanorods anchored on CNFs created a highly porous conductive network with active edge-plane exposure, enabling adsorption-controlled processes at low scan rates but diffusion-controlled processes at higher scan rates (Figure 4A) [47]. Electron microscopy images of CNF–NiCoSe_4_ composites, showing the uniform distribution of nanorods along the CNFs. The corresponding cross-bonded elemental mapping confirms the spatial distribution of Ni, Co, and Se, demonstrating successful integration of the metal selenide onto the carbon framework (Figure 4B). This dual-mode behavior underscores the importance of balancing adsorption affinity and electron transfer kinetics in hybrid systems.

MWCNT-based hybrids further demonstrate that synergistic interactions with metal oxides or dopants can alleviate the intrinsic drawbacks of CNTs, such as aggregation and limited selectivity [78,82,83]. The incorporation of Ce-doped ZnO not only improved electron transfer, but also reduced van der Waals bundling effects, thereby exposing additional electroactive sites [78]. Interestingly, while Apt functionalization of MWCNT-Au electrodes increased theoretical selectivity in buffer conditions, real-sample testing sometimes revealed higher sensitivity for the non-functionalized hybrid. This divergence highlights the complexity of biological matrices and suggests that hybrid design must be optimized with realistic sample environments in mind rather than relying solely on buffer-based validation.

Magnetic carbon-metal hybrids, such as Fe_3_O_4_ nanoparticles combined with CQDs, illustrate another design rationale by leveraging magnetic enrichment and rapid electron transfer [79]. These systems show strong signal amplification yet often lack sufficient validation in real samples. This gap reflects a broader issue in the field where many reported hybrids emphasize low detection limits, but do not systematically address matrix effects, stability under physiological conditions, or reproducibility across different fabrication batches. These indicates that the exceptional analytical performance of carbon-metal hybrids stems from complementary functions: conductive carbon matrices enabling fast charge transport and surface adsorption, and metallic domains driving catalytic oxidation of Trp. However, reproducibility, selectivity in multi-analyte systems, and validation in complex fluids remain unresolved challenges.

### 4.4. Trp-Sensing Electrodes Modified with Polymers

Polymers, particularly conducting types like polyaniline, polypyrrole, and PEDOT:PSS, are integral to electrochemical sensor design due to their tunable conductivity, biocompatibility, and ease of functionalization [84]. These polymers facilitate the creation of molecularly tailored microenvironments, enhancing selective recognition capabilities. Their inherent flexibility also supports integration into wearable platforms. However, challenges such as polymer degradation and batch-to-batch variability persist, underscoring the need for composite materials that incorporate carbon or metal additives to stabilize the polymer matrix.

Their integration with carbon nanomaterials such as MWCNTs has been shown to enhance conductivity and stability, while molecularly imprinted polymers (MIPs) further introduce analyte-specific selectivity [84,85]. Representative examples are summarized in Table 6. A critical evaluation of the reported polymer-based systems reveals several distinct mechanistic advantages. First, the incorporation of functionalized CNTs into polymeric matrices provides a dual benefit: the π-π interaction between the indole moiety of Trp and CNT sidewalls promotes efficient electron transfer, while polymeric backbones offer electrostatic interactions with the carboxyl group of Trp. This synergy accounts for the enhanced peak resolution and the suppression of surface fouling, as demonstrated in f-MWCNTs–polymer composites [86,87]. Such surface chemistry considerations are more decisive for long-term stability than the absolute sensitivity metrics that are often emphasized.

**Figure 5:**
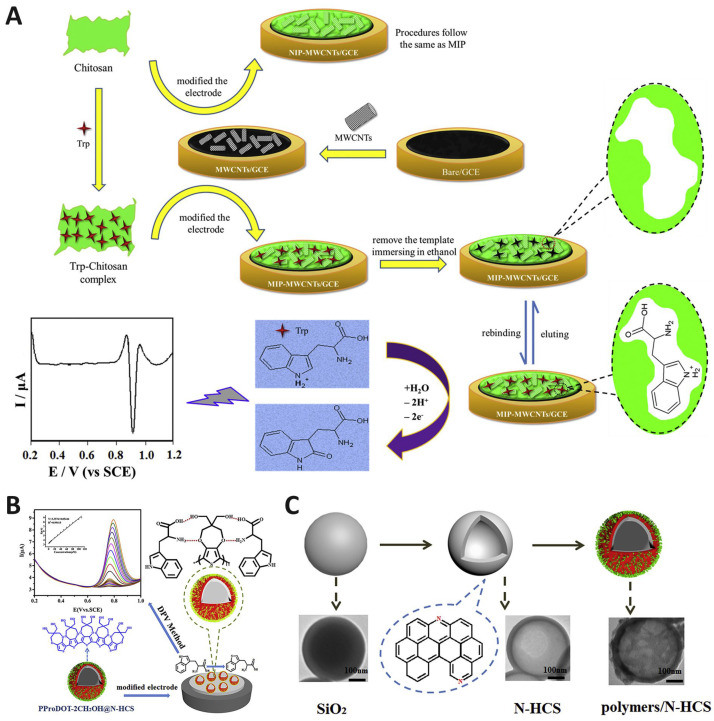
(**A**) Strategy for the fabrication of MIP−MWCNTs/GCE. Adopted with permission from Ref. [89]. Copyright Elsevier B.V., 2020. (**B**) Scheme of functionalized PProDOT@N-HCS electrodes, and (**C**) synthesis process of the functional microspheres. Adopted with permission from Ref. [92]. Copyright Elsevier B.V., 2020.

MIP-based architectures, while traditionally limited by poor conductivity, achieve higher analytical performance when combined with conductive supports such as MWCNTs. The success of MIPs arises from their ability to create molecular cavities that mimic the steric and electronic environment of Trp, as shown in Figure 5A [51,89]. This results in remarkable selectivity even in the presence of structurally similar interferents (AA, UA, DA, Tyr). Importantly, the challenge of template removal, which dictates the fidelity of the imprinted cavities, remains a bottleneck for practical translation. Thus, rather than only reporting LOD values, a more pressing criterion is the reproducibility of template removal and the maintenance of imprint fidelity under complex biological conditions.

Another promising avenue involves the integration of conducting polymers with functionalized carbon nanostructures. Poly(3,4-proplenedioxy thiophene) (PProDOT) is a derivative of a conducting polymer of poly(3,4-ethylenedioxythiophene) (PEDOT) with improved processibility. Introducing certain functional groups such as –OH and –SH can improve sensor sensitivity due to bonding interactions such as hydrogen bonding and π-π stacking between the analyte and functional groups (Figure 5B,C) [92]. Here, hydrogen bonding and π-π stacking interactions between the polymer functional groups and Trp introduce a level of molecular recognition that approaches that of biological receptors. These results suggest that rational functional group design within conductive polymers may rival or complement MIPs in selectivity while retaining superior conductivity.

In addition, strategies that shift the electro-oxidation potential of Trp into less-congested windows are particularly impactful [88,97]. The application of Schiff-base complexes on polymer-modified electrodes represents a strategic solution to minimize interference and memory effects by enabling Trp detection at lower potentials. This approach is conceptually different from sensitivity-driven optimization and highlights the importance of electrochemical window engineering in sensor design. In brief, polymer-modified Trp sensors embody a spectrum of strategies, ranging from imprinting-based molecular selectivity to functional group engineering and electrochemical potential modulation. While sensitivity and LOD values vary across studies, these are best contextualized by the underlying material strategy and application domain. Future directions should prioritize (i) robust template removal protocols in MIPs, (ii) rational functional group incorporation in conducting polymers for Trp-specific recognition, and (iii) electrochemical potential engineering to reduce biological interference. Only through such mechanistically informed design can polymer-based Trp sensors achieve a reproducibility, selectivity, and robustness suitable for clinical translation.

### 4.5. Trp-Sensing Electrodes Modified with Other Materials

Ionic liquids (ILs) and ionic liquid crystals (ILCs) offer a unique combination of ionic conductivity and ordered molecular orientation, making them promising modifiers in electrochemical sensor development [98]. ILs contribute to antifouling resistance and tunable selectivity, while ILCs provide enhanced structural organization. Despite these advantages, their high viscosity and limited mechanical stability necessitate their incorporation into composite matrices to optimize sensor performance. These properties provide a distinctive platform for charge transport, interfacial ion exchange, and structural stability, making ILC-based systems attractive for amino acid sensing. Representative examples are summarized in Table 7.

A mechanistic examination of ILC-assisted electrodes suggests that their contribution extends beyond conductivity enhancement. The ionized moieties within ILCs enable facilitated ionic shuttling through conductive matrices such as rGO sheets, thereby reducing charge transfer resistance and lowering oxidation potential. This is particularly relevant for the electrooxidation of Trp, where proton-coupled electron transfer pathways are sensitive to local ionic mobility. Moreover, the anisotropic molecular ordering of ILCs promotes preferential orientation of nanostructures (such as CNTs or metal nanoparticles) at the electrode interface, which may further increase electroactive surface area and electron transfer kinetics. This synergy accounts for the higher oxidation currents and improved stability reported in ILC-modified systems [98].

Another important consideration is the chemical environment modulated by IL. pH-dependent studies indicate that IL composites facilitate electrostatic interactions between the deprotonated carboxyl group of Trp and positively charged electrode interfaces, thereby reinforcing molecular recognition. This implies that ILCs not only enhance electron transfer, but also provide a favorable microenvironment for selective binding. The ability to integrate ILs with semiconducting oxides (e.g., SnO_2_ or Co_3_O_4_) or carbides (e.g., titanium carbide (TiC)) introduces additional catalytic sites and redox activity, highlighting the versatility of IL-based composites [99,100]. Despite these advantages, certain challenges remain. The intrinsic viscosity of IL, although useful for stabilizing ionic domains, may hinder diffusion of analytes at high loading rates. Furthermore, the reproducibility of IL ordering within nanocomposites is highly sensitive to electrode-preparation protocols, which may explain variations in analytical performance across different studies.

Recent years have witnessed growing interest in the integration of MXenes and metal-organic frameworks (MOFs) into electrochemical sensors for Trp detection (Table 8). Both materials exhibit tunable electrochemical properties, yet they achieve signal enhancement through distinct mechanisms. MXenes are 2D transition-metal carbides/nitrides characterized by metallic conductivity, high surface area, and surface-terminating groups (-O, -OH, -F) [102,103,104,105]. Their hydrophilic surface chemistry facilitates polymer integration and ionic liquid stabilization, enabling efficient electron transfer and strong noncovalent interactions with Trp. For instance, oxygen-containing functionalities on MXene nanosheets promote hydrogen bonding and electrostatic attraction with the carboxyl group of Trp, while conjugated polymers (such as PEDOT or PPy) further strengthen π–π interactions with the indole moiety [102]. This cooperative effect not only accelerates charge transfer, but also stabilizes MIP films, providing exceptional selectivity. Thus, MXene-based sensors typically deliver ultra-low detection limits and a wide LR, making them promising candidates for trace-level detection in complex food and biological matrices.

By contrast, MOFs contribute to sensing platforms through their crystalline porous structures and chemically tailorable frameworks [106,107]. Their ordered pore networks facilitate size-and shape-selective adsorption of Trp, while coordinatively unsaturated metal sites and functionalized linkers serve as catalytic hotspots. However, the intrinsically low conductivity of pristine MOFs is a critical limitation. This drawback is often overcome by coupling MOFs with conductive carbonaceous supports (rGO, CNTs) or with MXenes, generating hybrid systems where MOFs provide molecular sieving and enrichment, while conductive scaffolds ensure rapid electron transport. Such composites balance selectivity and conductivity, producing stable and reproducible responses in biological samples.

Despite their promise, both MXenes and MOFs face practical challenges. MXenes are prone to oxidative degradation in aqueous or ambient conditions, potentially compromising long-term stability. MOFs, while highly tunable, can suffer from structural collapse or leaching of metal ions under physiological conditions. Moreover, the fabrication protocols (etching, solvothermal synthesis, in situ polymerization) can be complex and difficult to standardize, raising concerns about reproducibility across laboratories.

### 4.6. Tryp-Sensing Electrodes Modified Nanomaterials

The electrochemical detection of Tryp remains less explored than that of Trp due to its low physiological abundance, high variability, and weaker oxidation signal. Trp, an essential amino acid and precursor of SR and melatonin, exhibits a strong and well-defined indole oxidation peak, making it reliably detectable on conventional electrodes. In contrast, Tryp requires highly sensitive, often nanomaterial-enhanced electrodes for detection. Its oxidation follows a quasi-reversible one-electron/one-proton mechanism, initiating at C_2_ of the indole ring and proceeding via secondary oxidation and hydroxylation at C_7_, which complicates mechanistic interpretation. Also, this quasi-reversible oxidation often causes electrode fouling that reduces sensor long-term stability [5]. These explain both the scarcity of literature on Tryp and the more concise coverage of this analyte compared with Trp in our review.

Table 9 summarizes the reported sensors for Tryp detection. Several strategies have been pursued to address the fouling challenge, including the use of MIPs to provide structural selectivity, nanomaterials (MWCNTs, AuNPs, carbon derivatives) to enhance conductivity and surface area, and enzyme-based systems (e.g., diamine oxidase (DAOx) or monoamine oxidase (MAOx) that leverage the enzymatic conversion of Tryp into electroactive products [35,108,109]. In most cases, hybrid architectures, such as polymer/Gr composites or enzyme/NPs-modified electrodes. They were essential to achieve adequate sensitivity and selectivity in complex matrices such as cheese, beverages, and wine.

Nevertheless, compared with the extensive advancements in Trp sensors, current Tryp-sensing technologies face several unresolved challenges. First, structural similarity to Trp and Tryp complicates selectivity, often requiring MIPs or enzymatic pathways to ensure discrimination [35,108]. Second, electrode fouling by polymeric oxidation products remains a major barrier to reproducibility and long-term stability [5,111]. Third, while enzyme-based sensors show promising selectivity, they often suffer from limited operational stability and batch-to-batch variability [109]. Finally, paper-based and disposable formats, though attractive for rapid screening, generally offer lower sensitivity compared with nanomaterial-modified electrodes [111,112].

The relative scarcity of reports compared with Trp sensors reflects both the lower historical prioritization of Tryp in biomedical sensing and the greater analytical complexity arising from its oxidation pathway and interference profile. This discrepancy underscores the need for systematic research into (i) anti-fouling electrode materials (e.g., conductive polymers with redox mediators, antifouling coatings), (ii) biorecognition strategies beyond MIPs and enzymes (such as Apt- or MOF-based selective adsorption), and (iii) miniaturized POC devices capable of multiplexed detection of biogenic amines [5,35,108,109,111,113]. In summary, while progress has been made using nanostructured electrodes and imprinting strategies, Tryp electrochemical sensing remains less mature than Trp. Bridging this gap will require mechanistically guided sensor designs that simultaneously overcome fouling, improve selectivity against structurally related interferents, and achieve robust performance in real sample matrices.

## 5. Trp, Tryp, and Their Roles in Oral Pathophysiology

The oral cavity reflects both local and systemic health, with Trp and its derivative, Tryp, playing pivotal roles in immune modulation, microbial homeostasis, and disease progression. Trp serves as a precursor to SR and melatonin, linking its metabolism to mood, circadian regulation, and inflammatory responses. The microbial and enzymatic conversion of Trp to bioactive compounds such as Tryp further influences oral and systemic physiology.

Saliva provides a non-invasive matrix for monitoring these metabolites, offering insight into oral diseases, including periodontal disorders and oral cancers, as well as systemic conditions [114,115]. The reliability of salivary Trp and Tryp as biomarkers depends on controlling pH, enzymatic activity, and potential interference from other biomolecules. Collectively, Trp and Tryp in saliva represent informative indicators of metabolic and immunological changes in the oral cavity. Understanding their dynamics not only advances the mechanistic understanding of oral pathophysiology, but also supports the development of diagnostic and prognostic strategies for oral and systemic health.

### 5.1. Oral Pathophysiology

In this section, we focus on how Trp and its derivatives mentioned earlier can be used to monitor oral pathophysiologies, from periodontal disease to various types of cancers. Here, we present some representative results to demonstrate the utility of Trp-related sensing and its associated disease linkages. In addition, we also discuss the important question of the temporal stability of the salivary microbiota in oral health.

Periodontal Disease: Melatonin is synthesized from Trp via SR as a precursor. It is classified as an indoleamine, which means it contains an indole ring (from Trp) and an amine group. Therefore, monitoring melatonin can be a surrogate for Trp. It has antioxidant and anti-inflammatory properties and is present in both saliva and gingival crevicular fluid (GCF). One study investigated the role of melatonin in periodontal health, focusing on its presence in saliva and gingival crevicular fluid (GCF) [116]. The study compared melatonin levels across 45 subjects categorized into healthy, gingivitis, and chronic periodontitis groups. They detected melatonin in both saliva and GCF, though GCF concentrations (mean: 1.54 pg/mL) were lower than salivary levels (mean: 2.17 pg/mL). Notably, melatonin levels in both fluids decreased progressively with disease severity, reaching their lowest concentrations in periodontitis patients (saliva: 0.07 pg/mL; GCF: 0.06 pg/mL), inversely correlating with clinical indices of inflammation and tissue destruction (*p*-value < 0.05). While no direct correlation between salivary and GCF melatonin levels was observed (*p*-value > 0.05), the study posits that reduced melatonin in diseased states reflects compromised anti-inflammatory defenses, potentially leading to increased oxidative damage and breakdown of periodontal tissues. However, both fluids reflect disease-associated melatonin depletion, emphasizing its therapeutic and diagnostic relevance in periodontal pathology. Melatonin is proposed as a biomarker for monitoring periodontal disease progression, though further studies with larger cohorts are needed to validate and standardize its clinical use.

Periodontal Inflammation: Another study explored the interplay between periodontal inflammation and Trp metabolism via the Kyn pathway, emphasizing its local (salivary) and systemic effects [19]. The study compared stage III periodontitis patients with healthy controls, analyzing saliva and serum for Kyn pathway metabolites, including Trp, Kyn, kynurenic acid (KynA), picolinic acid (PA), QA, and interleukin-6 (IL-6). These metabolites influence immune regulation and host-microbiome signaling and are largely produced by indoleamine 2,3-dioxygenase (IDO) in immune cells such as macrophages and dendritic cells. They found that salivary Trp, Kyn, KynA, IL-6, PA, and QA levels were significantly higher in periodontitis (*p*-value < 0.05) compared to healthy controls, alongside increased clinical indicators of disease severity (e.g., probing depth, clinical attachment loss, and bleeding on probing). Despite increased Trp and Kyn, the salivary Kyn/Trp ratio, a proxy for IDO activity, was significantly reduced, suggesting altered or dysregulated KP metabolism during periodontal inflammation, increased Trp release via inflammatory proteolytic degradation of the periodontium, and accelerated excretion of Trp via increased GCF in inflamed tissue.

Oral microbiota may contribute to this dysregulation. Microbial stimulation of IDO activity has been shown to enhance Trp metabolism, increasing Kyn levels in saliva [19]. Elevated salivary Trp and its metabolites have also been observed in diabetic patients with poor periodontal status, reinforcing the link between systemic inflammation, metabolic dysregulation, and Kyn pathway activation in the oral environment. Collectively, these findings suggest that salivary Trp and its downstream Kyn pathway metabolites reflect local immune activation and inflammatory burden in periodontal disease. Their non-invasive detectability and correlation with clinical indices highlight their potential utility as biomarkers for periodontal diagnosis, monitoring, and possibly prognosis.

Alzheimer’s Disease (AD): A 2023 study by Yang et al. investigated salivary metabolic changes and oral microbiota composition in AD, with a focus on Trp metabolism [18]. By analyzing saliva from AD patients and matched healthy controls under similar periodontal conditions, they identified significant shifts in salivary metabolites and bacterial profiles associated with AD. The study compared AD patients and healthy controls with matched periodontal conditions to analyze periodontal indices, salivary metabolites, and oral flora. AD patients exhibited significantly higher plaque indices and bleeding on probing, alongside distinct salivary metabolic profiles. Several Trp-derived metabolites—including 3-methyldioxyindole, indole-3-ethanol, and 3-methylindole—were reduced in the AD group. These compounds are typically produced by oral Gram-negative bacteria such as *Prevotella intermedia*, *Fusobacterium nucleatum*, and *Porphyromonas gingivalis*, which degrade Trp into indoles and phenolic compounds. However, AD patients showed reduced proportions of these bacteria, correlating with lower salivary levels of their Trp-derived metabolites (e.g., 3-methyldioxyindole). Paradoxically, this microbial shift coincided with increased systemic synthesis of neuroactive Trp derivatives, including SR, melatonin, and Tryp, compounds implicated in brain health.

Overall, the study highlights a disruption in Trp metabolism in AD saliva, likely driven by changes in oral microbial composition. These findings suggest that altered oral flora in AD may redirect Trp metabolism toward neuroprotective indoleamine synthesis (e.g., SR) rather than salivary excretion and bacterial degradation, potentially modulating AD progression. The study highlights saliva as a reservoir for AD biomarkers and underscores the role of oral microbiome-host metabolic crosstalk in neurodegenerative disease mechanisms.

Oral squamous cell carcinoma (OSCC): Saliva is a promising non-invasive source of biomarkers for the early detection of OSCC, which accounts for over 90% of oral cancers and remains a major cause of cancer mortality worldwide [117]. Despite the accessibility of the oral cavity, OSCC is often diagnosed late, contributing to its low five-year survival rate (~62%). Salivary metabolomic studies have identified several amino acids, including Trp, as promising biomarkers for OSCC detection. Among over 100 salivary biomarkers investigated, Trp is listed as a metabolite of interest. A second study using capillary electrophoresis-mass spectrometry (CE-MS) analyzed saliva from OSCC patients and healthy controls, identifying 499 metabolites, 25 of which discriminated OSCC from controls (*p*-value < 0.05–0.001) [118]. Trp was among the significantly elevated metabolites in OSCC saliva (*p*-value < 0.05), aligning with prior findings, where it also emerged as a shared biomarker [119]. Among the altered metabolites, Trp—a precursor to the neuroactive compound Tryp—was significantly increased in OSCC saliva. Other discriminatory metabolites included choline, cadaverine, and branched-chain amino acids-related compounds (valine, leucine, isoleucine, and 2-oxoisovaleric acid), all of which have roles in cancer-related metabolic pathways. Trp’s presence in the discriminatory metabolite panel underscores its potential role in OSCC-associated metabolic reprogramming. The study highlighted p-hydroxyphenylacetic acid—a Tyr metabolite linked to *Porphyromonas gingivalis*—as elevated in OSCC, suggesting oral microbiome interactions may indirectly influence aromatic amino acid pathways.

Breast Cancer: Salivary Trp levels are elevated in patients with breast cancer, correlating with tumor aggressiveness and malignancy, particularly in triple-negative and luminal B-like HER2-negative subtypes [114]. Increased salivary Trp was associated with advanced cancer stage, lymph node metastasis, poor differentiation, lack of HER2, ER, and PR expression, and high proliferative tumor activity. Trp levels declined post-tumor resection, reinforcing its association with disease presence. Limitations of the study include a small sample size, absence of Kyn pathway metabolite analysis, and lack of control for associated diseases, or dietary or metabolic confounders. Salivary Trp shows promise as a non-invasive biomarker for monitoring metabolic dysregulation in breast cancer.

Thyroid cancer: A study developed a robust ultra-high-performance liquid chromatography-high-resolution mass spectrometry (UPLC-HRMS) method to quantify salivary amino acids for early papillary thyroid carcinoma (PTC) diagnosis [120]. Significant alterations in 10 amino acids including Trp (*p*-value < 0.05) were found between PTC and healthy controls, and a biomarker panel achieved an area under the curve of 0.936 (sensitivity 91.2%; specificity 85.2%). Trp metabolism, driven by IDO overexpression in thyroid tumors, was implicated in tumor progression. Enhanced IDO activity depletes systemic Trp while increasing Kyn pathway metabolites, fueling tumor growth. Despite some limitations, salivary Trp is positioned as a promising non-invasive biomarker for early PTC detection.

Temporal Stability of the Salivary Microbiota: Saliva is increasingly recognized as a valuable, non-invasive medium for detecting biomarkers associated with both oral and systemic health. Beyond oral diseases, salivary biomarkers have been linked to systemic conditions including breast and thyroid cancers. The clinical application of salivary diagnostics depends on understanding the stability of salivary components over time. Belstrøm et al. conducted a systematic study to assess whether the composition of the salivary microbiota in orally healthy individuals fluctuates over short time periods [121]. Stimulated saliva samples were collected at 4-h intervals across a 24-h period and then again one week later. Using the high-resolution Human Oral Microbe Identification using Next Generation Sequencing (HOMINGS) technique, the bacterial profiles of 60 samples from five healthy subjects were analyzed and 477 microbial probes were identified. The results revealed minimal within-subject variation in microbial composition across both the 24-h and one-week intervals. While inter-individual differences were apparent, the microbiome of each individual remained remarkably stable over time. Dominant genera, such as *Streptococcus*, were consistently detected, while recognized periodontal pathogens like *Porphyromonas gingivalis* and *Aggregatibacter actinomycetemcomitans* were rarely identified, consistent with their low prevalence in oral health. These findings provide strong evidence that the bacterial composition of saliva is temporally stable in healthy individuals, supporting the use of microbial profiles as reliable diagnostic biomarkers.

### 5.2. Electrochemical Sensing Systems in Oral Hygiene and Disease Monitoring

Electrochemical biosensors have emerged as highly promising platforms for salivary diagnostics, offering non-invasive, rapid, and cost-effective detection of biomarkers associated with oral cancer and systemic health. Unlike invasive biopsies or costly imaging modalities, electrochemical sensing enables real-time molecular monitoring at the POC, particularly valuable in resource-limited settings.

Of particular interest are sensors targeting salivary metabolites such as Trp and its derivatives (e.g., Tryp and melatonin). These molecules not only reflect metabolic reprogramming and immune evasion in malignancies, but also serve as accessible indicators for personalized oral healthcare. Recent developments demonstrate impressive analytical sensitivity; however, a critical comparison reveals both opportunities and persistent barriers to clinical translation. For example, the application of nicking endonuclease signal amplification (NESA) on TiO_2_ electrodes achieved ultrasensitive DNA detection with femtomolar resolution, targeting oncogene-related transcripts in saliva [122]. Similarly, innovative host-guest systems employing Trp-linked DNA with methyl viologen-cucurbituril complexes enabled the selective amplification of cancer-associated signals, while nanomaterial-based platforms incorporating noble metal catalysts enhanced electron transfer and catalytic activity for robust signal amplification [123].

Electrochemical biosensors are particularly impactful in targeting salivary metabolites such as Trp and its derivatives, which play roles in metabolic reprogramming and immune evasion in oral cancers. This metabolite-centered approach complements the detection of canonical biomarkers, including interleukins (e.g., interleukin-1 beta (IL-1β)), tumor necrosis factor alpha (TNF-α), metabolites, and microRNAs, thereby expanding diagnostic capacity beyond single-marker strategies [124,125,126]. Advances in nanostructured electrodes, lab-on-chip (LOC) devices, and surface modification have further improved sensor selectivity and reproducibility, with multiplexed detection platforms enabling simultaneous monitoring of multiple salivary biomarkers [126]. A summary of the key findings is presented in Table 10.

While the reported LODs rival or surpass ELISA, major barriers to translation include: (i) variability in salivary composition, requiring robust normalization strategies; (ii) sensor stability, particularly under continuous intraoral exposure; and (iii) limited validation in large, diverse populations. Looking ahead, integration with AI-driven calibration, multi-marker sensing, and scalable 3D-printed microfluidic devices could bridge the gap between laboratory prototypes and routine clinical screening, potentially transforming early oral cancer diagnostics [13,127].

## 6. Challenges and Perspectives

Electrochemical biosensors for Trp and its metabolite Tryp hold considerable promise for the non-invasive monitoring of physiological and pathological conditions, including cancer, neurodegeneration, and oral diseases. While recent innovations in materials science and device engineering have pushed detection limits to picomolar levels and enabled multi-analyte capabilities, translating these advances into reliable, clinical-grade diagnostics remains a formidable challenge. Below, we outline the key scientific, technical, and translational barriers, along with emerging solutions, toward realizing robust, real-time Trp/Tryp biosensing platforms.

### 6.1. Biochemical and Electrochemical Challenges of Trp/Tryp-Sensing

Selectivity, Stability, and Electrode Durability: The electrochemical detection of Trp and Tryp remains inherently challenging due to their molecular structures and redox behaviors. Trp’s indole ring confers distinct electroactivity, but also predisposes the molecule to oxidative instability, particularly in acidic environments, where degradation by-products complicate signal interpretation and reduce reproducibility [20]. Additionally, the electrochemical potential windows of Trp and Tryp overlap with those of structurally related biomolecules, including SR and Tyr. This overlap generates convoluted oxidation peaks that impair selectivity, especially in complex biofluids such as saliva, where multiple electroactive interferents coexist [98]. Electrode fouling is another critical barrier. Nonspecific adsorption of proteins and metabolites on electrode surfaces leads to progressive signal drift and diminished sensitivity during repeated measurements.

Perspectives: Overcoming these challenges requires integrated, multi-pronged approaches that combine materials science and engineering, electrochemical innovation, and advanced data analytics. MIPs designed to bind indole derivatives or enzyme-modified electrodes with substrate specificity, provide promising routes to enhance molecular recognition and selectivity. On the signal analysis side, DPV coupled with multivariate statistical tools or machine learning-assisted signal deconvolution offers powerful strategies to resolve overlapping oxidation peaks, thereby improving interpretability [13,24,98].

The clinical significance of Tryp remains relatively underexplored, highlighting the need for systematic studies that link sensing performance to diagnostic outcomes. Addressing long-term stability will be essential for translation into real-world applications. Promising antifouling approaches such as polyethylene glycol coatings, self-cleaning surface modifications, and electrode regeneration techniques, show potential, but require refinement to achieve reproducible, long-duration operation in biofluids [128,129]. Future progress in this area will depend on the convergence of robust electrode design, antifouling surface engineering, and intelligent signal processing methods. Also, while Trp and Tryp are biologically relevant, their diagnostic utility remains underexplored, highlighting the need for systematic validation in large patient cohorts.

### 6.2. Sensing in Complex Biofluids: The Case of Saliva

Saliva as a Complex Diagnostic Medium: Saliva is increasingly recognized as a valuable diagnostic fluid for non-invasive monitoring; however, it is also one of the most challenging matrices for biosensing. Unlike plasma, saliva is a heterogeneous medium with fluctuating physical and biochemical properties. Its rheological characteristics, strongly influenced by mucins and glycoproteins, alter viscosity and affect analyte diffusion [114,115,117]. Additionally, the enzymatic environment of saliva, which contains proteases, amylases, and nucleases, can compromise biomarker stability. The oral microbiome contributes further metabolic variability, introducing by-products that may interfere with electrochemical signals. These factors are highly dynamic, modulated by circadian rhythms, diet, oral hygiene, and lifestyle factors such as tobacco and alcohol use [18,116].

Clinical conditions such as periodontal disease further reshape the salivary biochemical profile, complicating biomarker interpretation. Collection methodology adds another layer of complexity: differences between unstimulated passive drooling, expectoration, and swab-based sampling can lead to significant variations in analyte concentration and integrity. Moreover, stimulated saliva often altered flow rates and composition compared to unstimulated samples, influencing reproducibility and diagnostic reliability.

Perspectives: To mitigate these challenges, rigorous normalization protocols and standardized sampling strategies are essential. Harmonized protocols covering collection, storage, and preprocessing steps will be critical to ensure reproducibility across clinical studies [114,125,130]. Emerging technological innovations also hold promise. Microfluidic devices can improve sample handling and enable the precise metering of small volumes. Nanomaterial-based stabilization strategies and on-site preservatives can protect biomolecules from enzymatic degradation during storage and processing. Computational methods, including machine learning and multi-omics integration, are powerful tools to account for inter-individual variability and to extract consistent biomarker signatures across heterogeneous datasets [13].

Validation across diverse populations with different dietary, lifestyle, and genetic backgrounds remains a priority. Furthermore, systematic investigations of different oral niches—tongue, buccal mucosa, and salivary gland ducts—may provide complementary biomarker sources to enhance diagnostic robustness.

### 6.3. Biomarker Validation and Clinical Relevance

Emerging Biomarkers with Limited Validation and Clinical Standardization: The role of Trp and its metabolite Tryp as salivary biomarkers is still in its infancy. While Trp has received more attention, evidence linking Tryp to disease processes such as carcinogenesis and immune modulation via IDO activity remains largely associative rather than mechanistic [19]. This reflects a broader challenge in salivary diagnostics: distinguishing reliable disease-related signals from the inherent biochemical complexity of saliva. Many proposed metabolites, including Trp and Tryp, display limited disease specificity, raising the risk of false positives or negatives.

In particular, the observed correlations between Tryp and cancer remain preliminary and require further mechanistic clarification to establish true clinical relevance across both oral and systemic diseases. Furthermore, most candidate biomarkers for early detection have not yet been validated in large, demographically diverse cohorts. This gap emphasizes the need for reproducibility, meta-analyses, and standardized biomarker interpretation. Progress toward clinical translation will therefore require not only multi-institutional clinical studies, but also regulatory benchmarks, such as head-to-head comparisons with ELISA or other gold-standard assays, to objectively evaluate biosensor performance [10]. Equally important is the harmonization of biosensor outputs and clinical interpretation across laboratories to enable meaningful cross-study comparisons and build a robust evidence base.

Perspectives: Future progress hinges on addressing three interconnected challenges: validation, standardization, and regulatory alignment. First, biomarker validation will require large-scale, multi-center clinical trials involving >1000 patients to rigorously assess sensitivity, specificity, and performance in the presence of confounding conditions [131].

Second, moving beyond single biomarkers, emerging multi-omics approaches, including proteomics, transcriptomics, metabolomics, and metagenomics, hold promise for developing robust biomarker panels with higher diagnostic accuracy [115,119,120]. Collaborations with dental clinics for longitudinal sampling will be instrumental in generating reliable datasets that capture temporal dynamics and inter-patient variability. Standardization is equally essential. Consensus guidelines must be developed and consistently applied across the full diagnostic workflow, from patient preparation (e.g., fasting requirements) to saliva collection, storage, processing, and analysis. Uniform protocols and assay reproducibility are prerequisites for regulatory acceptance, especially in complex metabolic pathways such as Trp catabolism. Real-time detection technologies may further transform the field by enabling the dynamic monitoring of oral dysbiosis and its systemic effects, with potential implications for conditions ranging from chronic inflammation to neurodegeneration.

Finally, alignment with regulatory pathways remains a critical milestone. To date, no Trp/Tryp biosensor has advanced into formal pilot trials or regulatory submission processes such as ISO 13,485 certification or CE-marking [132,133]. Translation from bench to bedside will require early and proactive engagement with these frameworks, ensuring that device development adheres to safety, quality, and performance standards. Partnerships with both dental and medical clinics will be essential to integrate longitudinal sampling into validation pipelines, ultimately paving the way for clinical-grade adoption.

### 6.4. Sensor and Platform Engineering Barriers

Durable and Scalable Sensors for Microfluidic and CMOS Integration: Although electrochemical sensors are inherently compatible with microfluidic and CMOS-based platforms, their seamless integration remains technically challenging. Issues such as flow rate inconsistencies, reagent cross-contamination, and thermal instability during processes, particularly when multiple sample preparation steps, including cell sorting, lysis, nucleic acid isolation, and amplification, must be incorporated within a single compact device [125,126,127]. Achieving precise thermal regulation and maintaining channel integrity while integrating heterogeneous materials further increases system complexity. In parallel, sensor durability poses a significant bottleneck, as salivary proteins, degradative enzymes, and microbial contamination accelerate sensor degradation [124]. While antifouling coatings and the use of advanced nanomaterials such as Gr or QDs can enhance sensitivity and stability, they also introduce additional cost and fabrication complexity [69,82]. Furthermore, scaling these technologies for mass production, especially in POC and wearable formats, is hindered by challenges in miniaturization, such as reliable sub-50 micrometer feature printing.

Perspectives: To facilitate integration, modular approaches can be adopted, such as 3D-printed microfluidic modules, pre-loaded reagent cartridges, and magnetic bead chambers, which simplify device assembly and enhance reproducibility [79,127]. The performance of electrodes and microfluidic structures can be further improved through the use of advanced composite materials, metal additive manufacturing, and refined 3D-printing techniques including digital light processing and stereolithography. For scalable fabrication, strategies such as screen-printed electrodes, roll-to-roll manufacturing, and open-source platform designs offer cost-effective solutions while maintaining high throughput and reproducibility [55,109]. These approaches collectively provide a pathway to developing robust, modular, and economically viable biosensing platforms suitable for POC and wearable applications.

### 6.5. Simultaneous Multi-Analyte Detection

Peak Overlap and Signal Interference: The simultaneous detection of multiple analytes such as Trp, Tryp, SR, Tyr, and other amino compounds is essential for capturing comprehensive biochemical profiles relevant to disease diagnostics [17,73,75,113]. However, overlapping redox peaks create major obstacles, limiting both sensitivity and selectivity in conventional electrochemical sensing methods. These interferences are particularly problematic in multiplexed assays, where the accurate quantification of each analyte is crucial. However, the use of AI-assisted signal deconvolution techniques, such as machine learning-based classification and chemometric analysis, can effectively separate overlapping voltammetric signals, thus improving analytical resolution [134].

Perspectives: Several strategies have been developed to address this challenge:

1. Electrode Architecture: Advanced electrode designs, such as multiplexed microelectrodes, nanostructured surfaces, and spatially resolved electrode arrays, enable the independent and parallel detection of multiple analytes. Incorporating recognition elements such as MIP layers or enzyme-specific coatings further enhances selectivity and reduces cross-talk between signals [24,38].
2. Voltammetric Signal Deconvolution: Sophisticated electrochemical methods such as DPV, SWV, and EIS, when coupled with deconvolution algorithms, allow for the resolution of overlapping peaks and provide more accurate quantification [13,134].
3. Computational Approaches: Machine learning-based signal separation has emerged as a powerful tool to handle complex electrochemical datasets. Algorithms such as principal component analysis, support vector machines, and deep learning models can classify and quantify analytes from overlapping voltammetric signals with high accuracy. These tools enable discrimination between structurally related compounds and allow for robust analysis in complex biofluids.

Together, these advances highlight the feasibility of multiplexed sensing despite intrinsic biochemical challenges, paving the way for practical multi-analyte biosensors capable of real-world diagnostic use.

### 6.6. AI and Smart Biosensing

Machine Learning for Enhanced Interpretation: AI and machine learning algorithms hold transformative potential for refining the accuracy and reliability of biosensor outputs [134,135]. By learning from large volumes of electrochemical data, these models can decode subtle variations in current-voltage signatures, peak shapes, and temporal signal dynamics. Importantly, AI can also account for confounding biological and lifestyle factors, such as inflammation triggered by smoking, dietary variability, or circadian rhythms, that often obscure Trp/Tryp biosensor signals. For example, machine learning models have been shown to classify and quantify Trp concentrations in saliva with higher reproducibility than conventional calibration-based methods, reducing error margins caused by electrode fouling or environmental variability [134].

Despite these promising results, the performance of AI systems remains heavily dependent on the availability of high-quality, annotated datasets. Current limitations in dataset size, diversity, and metadata annotation hinder robust model training and generalization across different biosensor platforms and patient cohorts. To overcome these challenges, collaborative data-sharing consortia and standardized data acquisition protocols are urgently needed. Strategies such as transfer learning and federated learning also offer powerful means to leverage existing datasets across institutions while preserving data privacy and improving model generalization [136].

Perspectives: Several strategic directions are recommended to fully harness AI in Trp/Tryp biosensing:

1. High-Quality Dataset Development: Building open-access, annotated databases of electrochemical sensor responses across diverse populations, biological matrices, and disease states should be prioritized. Such databases will underpin robust and generalizable predictive modeling [134].
2. Standardization of Protocols: Harmonized standards for sample preparation, sensor calibration, and data logging will improve reproducibility and enable interoperability across biosensor platforms.
3. Feature Engineering and Multi-Omics Integration: Beyond raw voltammetric or amperometric data, AI models can incorporate engineered features (e.g., peak ratios, charge transfer kinetics, response times) along with contextual metadata (age, sex, circadian cycles). Integration with multi-omics datasets (genomics, proteomics, metabolomics) will allow biosensors to capture a more holistic biomarker signature and improve disease prediction accuracy [136].
4. Edge Computing and Real-Time Decision Support: Embedding lightweight AI algorithms into portable or wearable biosensors enables on-device signal processing with minimal latency and enhanced data privacy. Such approaches can enable the real-time monitoring of Trp/Tryp fluctuations, critical for continuous health tracking.
5. Adaptive Self-Calibration: AI can dynamically adjust for baseline shifts, electrode degradation, and environmental variations, extending sensor lifespan and improving reliability in long-term applications.
6. Explainable and Regulatory-Compliant AI: For clinical adoption, models must not only be accurate, but also interpretable. Explainable AI ensures that decision pathways are transparent, facilitating clinician trust and regulatory approval while minimizing risks of false positives or negatives.
7. Secure Data Handling: Encryption and adherence to privacy standards are essential for safeguarding sensitive health data, maintaining user trust, and supporting integration into personalized healthcare ecosystems.

In summary, while AI-augmented biosensing for Trp/Tryp remains at an early stage, initial demonstrations highlight substantial improvements in accuracy, robustness, and adaptability. With coordinated efforts in dataset generation, methodological standardization, and the integration of multi-omics and contextual information, AI has the potential to transform electrochemical Trp/Tryp biosensors into powerful, clinically relevant tools for real-time biochemical monitoring.

### 6.7. Toward POC and Home-Based Monitoring

Accessible Real-Time Personalized Health Tracking: Emerging trends in real-time, personalized health monitoring include the development of smartphone-compatible sensors, screen-printed electrochemical platforms, and wearable biosensors capable of continuous, at-home data acquisition. Integration with Bluetooth connectivity, cloud computing, and AI-driven applications has the potential to transform chronic disease management and facilitate early detection of oral potentially malignant disorders [134,136]. However, cost and accessibility remain significant challenges, particularly in low-resource settings. The reliance on advanced materials such as quantum dots and the expense of analyzers limit widespread adoption. To overcome these barriers, scalable fabrication methods including roll-to-roll printing, as well as the use of open-source hardware and sustainable materials, will be critical for democratizing biosensing technologies and ensuring equitable access to advanced diagnostic tools.

Perspectives: Addressing challenges in POC and home-based monitoring requires a multi-pronged strategy. Fully integrated, disposable LOC systems should combine sample handling, filtration, mixing, detection, and waste management within a single device [126]. Advances in 3D-printing, soft lithography, and paper-based microfluidics are enabling low-cost POC diagnostics, as exemplified by COVID-19 antigen tests and glucose strips. Non-invasive or minimally invasive sampling methods using saliva, sweat, breath, tears, or interstitial fluid via microneedle patches enhance user compliance and safety, particularly for chronic disease monitoring. Wearable sensors such as epidermal patches and smartwatches are central to this transformation. Automated calibration, internal standards, and drift-compensation algorithms ensure long-term accuracy and reduce reliance on professional maintenance. A robust sensor design that is capable of withstanding variable temperatures, humidity, and mechanical stress is essential for reliable performance in real-world environments. Coupling biosensors with AI-driven wellness recommendations, medication reminders, and lifestyle interventions transforms passive monitoring into active preventive health management.

## 7. Conclusions

Over the past decade, the electrochemical sensing of Trp and its key metabolite Tryp has advanced significantly, driven by innovations in electrode materials, nanostructures, and surface engineering. Carbon-based platforms, including CNTs, Gr derivatives, and hybrid nanocomposites, have played a central role in lowering detection limits while simultaneously improving sensor stability, reproducibility, and operational lifetime. These advances have enabled the reliable monitoring of indole-derived biomarkers in complex biological matrices such as saliva, serum, and urine, positioning electrochemical biosensors as practical tools for real-time molecular diagnostics. Despite these technical achievements, several translational challenges remain. The structural similarity of Trp and Tryp to other aromatic metabolites continues to challenge selectivity, and matrix effects from saliva or serum can further complicate accurate quantification. Variability in biofluid composition due to circadian rhythms, dietary intake, or individual physiology necessitates robust normalization strategies. Additionally, standardized validation protocols are lacking, and most studies are confined to proof-of-concept demonstrations under controlled laboratory conditions, with limited assessment in real-world patient cohorts.

Looking ahead, the next generation of biosensors must move beyond single-analyte detection to develop multiplexed, pathology-specific platforms capable of simultaneously monitoring Trp, Tryp, and downstream metabolites such as SR, melatonin, and Kyn. Such multi-parametric sensing approaches would enable a systems-level understanding of metabolic and neurochemical networks, providing valuable insights for precision diagnostics in oncology, psychiatry, and neurodegenerative disorders. Furthermore, the convergence of advanced nanomaterials, bioengineering strategies, and digital health technologies is expected to transition Trp- and Tryp-based sensing from experimental prototypes into clinically actionable tools, a transformation that will require not only continued materials innovation, but also rigorous clinical validation, scalable and reproducible manufacturing, and interdisciplinary collaboration across chemistry, engineering, and medicine. Ultimately, addressing these challenges holds the potential to redefine molecular health monitoring, facilitating early disease intervention, personalized therapy, and broader global healthcare accessibility; in summary, while significant progress has been made, realizing the full translational potential of Trp and Tryp biosensors will demand coordinated efforts spanning technological, biological, and clinical domains.

## Figures and Tables

**Table 1:** Concentration of Trp in Biofluids.

Biofluids	Conditions	Concentrations (µM)	References
Saliva	Oral squamous cell carcinoma	3.81 ± 0.62	[30]
Saliva	Control	4.4	[31]
Urine	Autism	36.47 ± 6.51	[32]
Urine	Control	69.63 ± 9.98	[32]
Plasma	Major depression	34.3 ± 1.9	[33]
Plasma	Control	46.6 ± 2.7	[33]

**Table 2:** Performance of gold-modified electrodes for Trp sensing.

Functionalized Electrode	Technique	LOD (nM)	LR (µM)	Real Sample	Ref.
AuNPs-rGO-Cr.6/GCE	SWV	480	0.l–2.5	Human blood serum	[45]
AuNPs/FSN/SPCE	DPV, EIS	0.01	6 × 10^−5^–0.250	Human blood serum	[52]
DGNs/Fe_3_O_4_@SiO_2_/DABCO/SPCE	DPV	0.002	7.0 × 10^−6^–0.2	Human blood serum	[53]
Au-PDNs/GCE	DPV	0.10	160–280	N/A	[54]
Apt-MWCNTs/AuSPE	CC-PSA	0.0049	1.0 × 10^−3^–0.2	Human blood serum, milk, saliva, urine	[55]
Apt-MWCNTs/AuE	CC-PSA	0.064	1.0 × 10^−4^–300	Human blood serum, milk, saliva, urine	[49]
AuNPs-MPC/GCE	DPV	24	101,000	Human blood serum	[56]
AuNP/EGPU	DPV	53	0.6–2.0	Synthetic urine	[57]
AuNP/rGO/P-Arg/GCE	DPV	100	10–100	Human urine	[58]

**Table 3:** Performance of metal- and metal-oxide-modified electrodes for Trp detection.

Functionalized Electrode	Techniques	LOD (nM)	LR (µM)	Real Sample	Ref
Al-Gr-Cu/GCE	DPV	9	1–1000	Urine	[15]
AgY/CPE	DPV	6.3	0.02–1.2	Milk, wheat flour	[43]
Ag-TiO_2_ NPs/GCE	Amperometry	3	10–220	Egg white	[59]
WO_3_/GCE	DPV	4.4	0.02–2000	Curd, egg, milk	[60]
PT-ZnO/GCE	SWV	8.5	0.4–200	Peanut extract	[61]
CeO-ZnO/CPE	DPV	10	0.02–25	Urine	[62]

**Table 4:** Performance of carbon derivative-based and composite electrodes for Trp detection.

Functionalized Electrode	Technique	LOD (nM)	LR (µM)	Real Sample	Ref.
CHS/rGO/GCE	DPV	2	0.05–175.8	Urine	[14]
POM-rGO/GCE	SWV	0.002	1 × 10^−5^–1 × 10^−3^	Human blood serum	[16]
nano-CeO_2_/rGO/GCE	SDLSV	6	0.01–10	Human blood serum, urine	[44]
Pd-Cu@Cu_2_O/rGO/GCE	DPV	1.9	0.01–40	Milk, urine	[46]
AgNPs/GO/GCE	DPV	2	0.01–800	Human blood serum, pharmaceutical	[67]
SGr-Bi_2_S_3_/SPCE	DPV	4	0.01–120	N/A	[68]
rAu-PtNPs/GQDs/GCE	SWV	0.3	0.001–0.1	Milk	[69]
TiO_2_/rGO/CPE	DPV	0.4	0.1–120	Huma blood serum, urine	[70]
MnCo_2_O_4_-rGO/GCE	Amperometry	1	0.004–112.9	Milk	[71]
N-Gr/CuCo_2_O_4_/CPE	DPV	4.1	0.01–3	Human blood serum, pharmaceutical, urine	[72]
MnWO_4_/rGO/GCE	DPV	4.4	0.001–120	Milk	[50]
ZrO_2_-CuO-CeO_2_/Gr/CPE	DPV	5.32	0.009–193	Human plasma, urine	[73]
AgM/rGO/GCE	Amperometry	5.7	0.02–147	Milk, oats	[74]
CuO-CeO_2_-rGO-MWCNT GCE	DPV	7.3	0.01–13.5	Human blood serum, urine	[75]
Co_3_O_4_/rGO/GCE	LSV	260	1–800	Amino acid	[76]

**Table 5:** Performance of hybrids electrodes for Trp detection.

Functionalized Electrode	Technique	LOD (nM)	LR (µM)	Real Sample	Ref.
NiCoSe_4_-CNF/GCE	Amperometry	0.68	0.005–0.095	Human blood serum, milk, tomato juice	[47]
Ce-ZnO/f-MWCNT/GCE	DPV	1.	0.01–0.1	Human blood, milk	[78]
MagNPs/CQDs/GCE	DPV	4.2	0.05–13.5	N/A	[79]
Ni NPS/N-C/CPE	SDLSV	6	0.01–80	Amino acid, human blood serum	[80]
MWCNT/Mg-Al-CO_3_/CPE	LSV	6.85	3–1000	Human blood serum, milk	[81]

**Table 6:** Performance of polymer-based composite electrodes for Trp detection.

Functionalized Electrode	Technique	LOD (nM)	LR (µM)	Real Sample	Ref.
MIP/CS/MWCNTs/GCE	DPV	500	1–300	Milk	[84]
OD/f-MWCNTs/p-AMT/GCE	Amperometry	0.54	0.025–0.3	Blood human serum	[86]
Schiff base complex NPs/AuE	EIS	0.78	0.004–0.06	Human blood serum	[88]
MIP-MWCNTs/GCE	SDLSV	1	0.002–100	Human blood serum	[89]
MIP@SiO_2_@PVP@AuNPs/GrE	LSV	300	1–350	Pharmaceutical	[51]
AuNPs/MWCNTs-Chit/SPE	DPV	1	0.003–100	Human blood serum	[90]
FC/CS-MWCNTs/GPE	DPV	3.7	1–200	Human blood serum, urine	[91]
PProDOT@N-HCS/GCE	DPV	8.3	1–70	Human blood serum	[92]
MIP/ABPE	SDLSV	8	0.01–100	Amino acid, human blood serum	[93]
PVP-Gr/GCE	SDLSV	10	0.06–100	Amino acid, human blood serum, urine	[94]
NiMn-LDH@PLL/GCE	DPV	52.7	0.1–130	Amino acid, urine	[95]
PEDOT:PSS/Gr/GCE	DPV	1.5	0.1–1000	N/A	[96]

**Table 7:** Performance of IL-based and composite electrodes for Trp detection.

Functionalized Electrode	Technique	LOD (nM)	LR (µM)	Real Sample	Ref.
rGO/ILC/CNT/Fe-Zn/GCE	DPV	1.58	0.008–30	Human blood serum	[98]
IL/SnO_2_-Co_3_O_4_@rGO/CPE	CV, DPV	3.2	0.02–6	Human blood serum, pharmaceutical, urine	[99]
IL/TiC/GCE	DPV	53	0.5–500	Milk, urine	[100]
MWCNT@IL/CPE	CV	2300	5–1000	Amino acid, human blood serum	[101]

**Table 8:** Performance of MXene-modified electrodes for Trp detection.

Functionalized Electrode	Technique	LOD (nM)	LR (µM)	Real Sample	Ref.
MIP/PEDOT/TiC/IL/GCE	DPV	2.09 × 10^−4^	1 × 10^−6^–100	Milk	[102]
MO/TiC/GCE	DPV	15	0.01–120	Egg, urine	[103]
TiC/GCE	DPV	309	0.5–20.1	Cheese, milk, yogurt	[104]
IL-TiC/GCE	DPV	0.06	0.001–240	Amino acid, urine	[105]
L-Lys-Ni Zn-MOF/Fc-MWCNTs/GCE	DPV	400	2–100	Milk	[106]

**Table 9:** Performance of different functionalized electrodes for Tryp detection.

Functionalized Electrode	Technique	LOD (nM)	LR (µM)	Real Sample	Ref.
GCE	SWV	0.80	0.047–0.54	Banna, cheese, sausage, tomato	[5]
PMU/PGE	DPV	329	0.5–40	Human blood serum	[17]
CS-MWCNTs/GCE	Amperometry	41.7	0.06–30	Cheese, lactobacillus beverage	[35]
MIP/HA-MWCNTs/PPy-SG/GCE	Amperometry	74	0.09–70	Cheese, lactobacillus beverage	[108]
DAOx/PtNP/SPCE	Amperometry	250	0.53–72	Cheese	[109]
MAOx/PtNP/SPCE		210	0.39–76		
DAOx/PVF/GO/SPCE	Amperometry	1400	6–340	Cheese	[110]
MAOx/PVF/GO/SPCE		1800	2.4–120		
CB-PCL/TPE	Amperometry	3200	10–75	Cheese, tap water	[111]
PTB/GS	SWV	12	0.05–0.9	Cheese	[112]
CPE-NiPC	SWV	0.85	0.0025–0.020	Wine	[113]

**Table 10:** Comparative performance of electrochemical biosensors for salivary detection of oral cancer biomarkers and metabolites.

Biomarkers	Strategy	Electrodes	LOD	Clinical Relevance	Ref.
ORAOV1 DNA	NESA	DNA/ITO	0.35 pM	Novel oncogene, linked to OSCC progression	[122]
Trp derivatives	Host-guest recognition	Metal catalysts/GCE	3 fM	Sensitive biosensor for ORAOV1/detection of oral tumor biomarker	[123]
IL-1β, TNF-α	Multiplexed electrochemical sensor	Hybrids/SPCE	0.38 pg/mL, 0.85 pg/mL	Inflammatory cytokines, validated in oral cancer	[124]
Uric acid	Wearable mouthguard	Enzyme integrated electrode	4.88 µM	Reflects metabolic stress in oral environment	[125]
OSCC mRNA panel	LOC system	Microfluidic DNA device	0.1 pg	Early diagnosis through multi-marker RNA	[126]

## Data Availability

Data are contained within the article.

## Abbreviations

AA	Ascorbic acid
ABPE	Acetylene black paste electrode
AD	Alzheimer’s disease
AgM	Silver molybdate
AI	Artificial intelligence
Apt	Aptamer
AuE	Gold electrode
AuNPs	Gold nanoparticles
AuSPE	Gold screen-printed electrode
Bi_2_S_3_	Bismuth sulfide
CB	Carbon black
CC-PSA	Constant-current potentiometric stripping analysis
CHS	Copper hexahydroxystannate
CMOS	Complementary metal oxide semiconductor
CNF	Carbon nanofiber
CPE	Carbon paste electrode
CQDs	Carbon quantum dots
CS	Chitosan
CV	Cyclic voltammetry
DA	Dopamine
DALYs	Disability-adjusted life years
DAOx	Diamine oxidase
DGNs	Dendritic gold nanostructures
DPV	Differential pulse voltammetry
EGPU	Modified graphite-polyurethane composite electrode
EIS	Electrochemical impedance spectroscopy
ELISA	Enzyme-linked immunosorbent assays
FC	Ferricyanide
Fc-MWCNTs	Ferrocene-modified multiwalled carbon nanotubes
Fe_3_O_4_@SiO_2_/DABCO	Magnetic double-charged diazoniabicyclo [2.2.2] octane dichloride silica hybrid
f-MWCNTs	Functionalized MWCNTs
FSN	Amine-functionalized silica nanoparticle
GCE	Glassy carbon electrode
GCF	Gingival crevicular fluid
GO	Graphene oxide
GPE	Graphite paste electrode
GQDs	Graphene quantum dots
Gr	Graphene
GrE	Graphite electrode
GS	Graphite sheets
HA-MWCNTs	Hyaluronic acid-multi-walled carbon nanotubes
HOMINGS	Human Oral Microbe Identification using Next Generation Sequencing
HPLC	High-performance liquid chromatography
IDO	Indoleamine 2,3-dioxygenase
IL	Ionic liquid
IL-1β	Interleukin-1 beta
IL-6	Interleukin-6
ILCs	Ionic liquid crystals
ITO	Indium tin oxide
Kyn	Kynurenine
KynA	Kynurenic acid
L-Lys-Ni	L-Lysine-functionalized nickel
LOC	Lab-on-chip
LOD	Limit of detection
LR	Linear range
LSV	Linear sweep voltammetry
MAOx	Monoamine oxidase
MIPs	Molecularly imprinted polymers
MnCo_2_O_4_-rGOs	Manganese cobaltite entrapped rGOs
MO	Methyl orange
MOF	Metal-organic framework
MWCNTs	Multi-walled carbon nanotubes
NaBH_4_	Sodium borohydride
NESA	Nicking endonuclease signal amplification
N-Gr	Nitrogen-doped graphene
N-HCS	Nitrogen-doped carbon hollow spheres
Ni NPS/N-C	Nickel nanoparticles/nitrogen-carbon nanohybrid
NiMn-LDH@PLL	NiMn-layered double hydroxide@poly-l-lysine
NiPC-CPE	Nickel phthalocyanine modified carbon paste electrode
NIPs	Non-imprinted polymers
NPs	Nanoparticles
OD	1,8-ocatane diamine
OSCC	Oral squamous cell carcinoma
PA	Picolinic acid
p-AMT	Poly(5-amino-2-mercapto-1,3,4-thiadiazole)
P-Arg	Poly(L-arginine)
PCL	Polycaprolactone
PDN	Polydopamine nanospheres
PEDOT	Poly(3,4-ethylenedioxythiophene)
PEDOT:PSS	Poly(3,4-ethylenedioxythiophene)-poly(styrenesulfonate)
PGE	Pencil graphite electrode
PMU	Poly-murexide
POC	Point-of-care
POM	Polyoxometalate
PProDOT	Poly(3,4-proplenedioxy thiophene)
PPy-SG	Polypyrrole-sulfonated graphene
Pt	Platinum
PT	Polythiophene
PTB	Poly(toluidine blue)
PTC	Papillary thyroid carcinoma
PVF	Polyvinylferrocene
QA	Quinolinic acid
rAu-PtNPs	Rod gold-platinum nanoparticles
*R_ct_*	Charge transfer resistance
rGO	Reduced graphene oxide
SDLSV	Second derivative linear sweep voltammetry
SERS	Surface-enhanced Raman scattering
SGr	Sulfur-doped graphene
SPCE	Screen printed carbon electrode
SR	Serotonin
SWV	Square wave voltammetry
TiC	Titanium carbide
TiO_2_	Titanium dioxide
TNF-α	Tumor necrosis factor alpha
TPE	Thermoplastic electrode
Trp	Tryptophan
Tryp	Tryptamine
Tyr	Tyrosine
UA	Uric acid
UPLC-HRMS	Ultra-high-performance liquid chromatography-high-resolution mass spectrometry
